# The need for home care physicians in Japan – 2020 to 2060

**DOI:** 10.1186/s12913-020-05635-2

**Published:** 2020-08-15

**Authors:** Hiroyoshi Iwata, Masato Matsushima, Takamasa Watanabe, Yoshifumi Sugiyama, Kenichi Yokobayashi, Daisuke Son, Yoshinao Satoi, Eriko Yoshida, Sumiko Satake, Yuki Hinata, Yasuki Fujinuma

**Affiliations:** 1 Division of Clinical Epidemiology, Research Center for Medical Sciences, The Jikei University School of Medicine, 3-25-8, Nishi-Shimbashi, Minato-ku, Tokyo, 105- 8461 Japan; 2 Kita-adachi Seikyo Clinic, Tokyo Hokuto Health Cooperative, Tokyo, Japan; 3 Home Care Clinic, Hiroshima, Japan; 4 Department of Medical Education Studies, International Research Center for Medical Education, Graduate School of Medicine, The University of Tokyo, Tokyo, Japan; 5 Kuji Clinic, Japanese Health and Welfare Co-operative Federation, Kawasaki, Japan; 6 Kawasaki-Kyodo Hospital, Japanese Health and Welfare Co-operative Federation, Kawasaki, Japan; 7 Department of Fundamental Nursing, The Jikei University School of Nursing, Tokyo, Japan; 8 Centre for Family Medicine Development, Japanese Health and Welfare Co-operative, Tokyo, Japan

**Keywords:** Home care medicine, Aged society, Health care workforce, Future prediction analysis

## Abstract

**Background:**

Japan faces the most elderly society in the world, and the Japanese government has launched an unprecedented health plan to reinforce home care medicine and increase the number of home care physicians, which means that an understanding of future needs for geriatric home care is vital. However, little is known about the future need for home care physicians. We attempted to estimate the basic need for home care physicians from 2020 to 2060.

**Methods:**

Our estimation is based on modification of major health work force analysis methods using previously reported official data. Two models were developed to estimate the necessary number of full-time equivalent (FTE) home care physicians: one based on home care patient mortality, the other using physician-to-patient ratio, working with estimated numbers of home and nursing home deaths from 2020 to 2060. Moreover, the final process considered and adjusted for future changes in the proportion of patients dying at home. Lastly, we converted estimated FTE physicians to an estimated head count.

**Results:**

Results were concordant between our two models. In every instance, there was overlap of high- and low-estimations between the mortality method and the physician-to-patient method, and the estimates show highly similar patterns. Furthermore, our estimation is supported by the current number of physicians, which was calculated using a different method. Approximately 1.7 times (1.6 by head count) the current number of FTE home care physicians will be needed in Japan in the late 2030’s, peaking at 33,500 FTE (71,500 head count). However, the need for home care physicians is anticipated to begin decreasing by 2040.

**Conclusion:**

The results indicate that the importance of home care physicians will rise with the growing elderly population, and that improvements in home care could partially suppress future need for physicians. After the late 2030’s, the supply can be reduced gradually, accounting for the decreasing total number of deaths after 2040. In order to provide sufficient home care and terminal care at home, increasing the number of home care physicians is indispensable. However, the unregulated supply of home care physicians will require careful attention in the future.

## Background

Japan has a ballooning elderly population which is the largest in the world. This super ageing society is expected to continue to grow [1, 2]. Projections for 2035 estimate 37,820,000 people aged 65 and above, or 33% of the total population, with further increases to 39.9% in 2060, while the national population has been decreasing since 2008 [3]. Even at present, Japan faces innumerable problems associated with ageing, and the medical field is no exception, particularly in the areas of geriatric and terminal care. One of the most important issues is how and where elderly people spend and end their lives.

The *Japanese* Ministry of Health, Labour and Welfare in Japan (MHLW) has proposed an unprecedented health policy called the “*Regional Healthcare Vision*” [4], to facilitate home medical care, allowing patients to end their lives at home or in a nursing home rather than a hospital. Recently, home death has been emphasized as a way to improve relationships with caregivers and maintain positivity among terminally ill cancer patients [5]. Further, home death is preferred by the majority of patients, caregivers, and the general public in many countries [6]. According to a survey of people aged 55 and above conducted by the Cabinet of Japan [7], more than half (54.6%) of Japanese citizens hoped to die at home, whereas only 13.2% of all deaths in 2017 were at home. These data imply that end of life care in Japan may not be sufficiently patient-centered. In addition, Japan tends to have the longest hospitalizations in the world [8]. If the trend of hospital terminal care continues, rising medical and welfare costs will require vast national expenditures.

In response, the MHLW created the *Regional Healthcare Vision* plan, adjusting and improving various community health systems. The MHLW promotes efficient hospital bed usage, restricts increases in hospital beds, and emphasizes home-visit medical care and nursing homes to encourage elderly people to stay out of hospitals. As the world’s fastest ageing country, Japan’s policy of promoting home medical care instead of building additional care facilities is unique.

Projecting the home care physician workforce is a significant task. However, we found only a single study by Matsushima et al., and an online news blurb summarizing its estimations for 2025, with poor data considerations [9, 10]. Forecasting needs and training home care physicians have received little attention for several reasons. First, Japan’s home care medicine and education systems are still developing. A survey reported that about half of clinics providing home care (47.3%) do not oversee home death, and only provide care to outpatients [11]. Furthermore, the systems associated with home care are complicated; both internists and general practitioners provide care, and there are four types of associated clinics and hospitals, with variation among nursing homes. Further, among full-time home care physicians, contributions to home care medicine vary because some provide care for out- or in-patients. In addition, there are both full-time and part-time physicians involved in home care medicine. Hence, this study aims to estimate the future minimum number of full-time equivalent (FTE) home care physicians required to care for patients at home or in nursing homes, applying two major workforce analysis methods: health needs and workforce-to-population ratio [12].

## Methods

In this study, we defined home care physicians as physicians who visit patients at home or in nursing homes and provide home care medicine. One FTE physician is defined as one full-time physician’s contribution to home care medicine; it does not include time not spent providing home care medicine, such as seeing out- or in-patients. Time spent providing home care medicine by part-time physicians was converted to FTE, using the proportion of the part-time physician’s average weekly or monthly time spent providing home care medicine to a full-time physician’s weekly or monthly working time at individual institutions.

Formula 1

$$ Projected\ annual\ number\ of\ home\ care\ physicians=\frac{Projected\ annual\ number\ of\ home\ and\ nursing\ home\ deaths\ }{Mortality\ \left( one\  year\right)\times Annual\ average\ number\ of\ patients\  per\  physician} $$

Formula 2

$$ Proje cted\ annual\ number\ of\ home\ care\ physicians= Home\ care\ \mathbf{physician}-\mathbf{to}-\mathbf{patient}\  ratio\times Proje\mathrm{c} ted\  annual\ home\ or\ nursing\ home\ death\ number $$

We employed two methods of analysis after calculating the annual number of home care patient deaths in order to estimate FTE physicians. The first is the mortality method, in which annual mortality among home care patients is used to estimate the total annual number of home care patients, enabling us to project the annual number of home care physicians by dividing by the average number of patients per physician. The second is the physician-to-patient ratio method, which applies the ratio of home care physicians and home or nursing home deaths (Additional file 1). In the mortality method, baseline home care physician data was from physicians who visit patients at home or in nursing homes, mainly at home care support clinics (HCSCs). However, in the physician-to-patient ratio method, data was from physicians who visit patients at home or nursing in homes, including many types of clinics and hospitals. (Additional file 2).

**Fig. 1:**
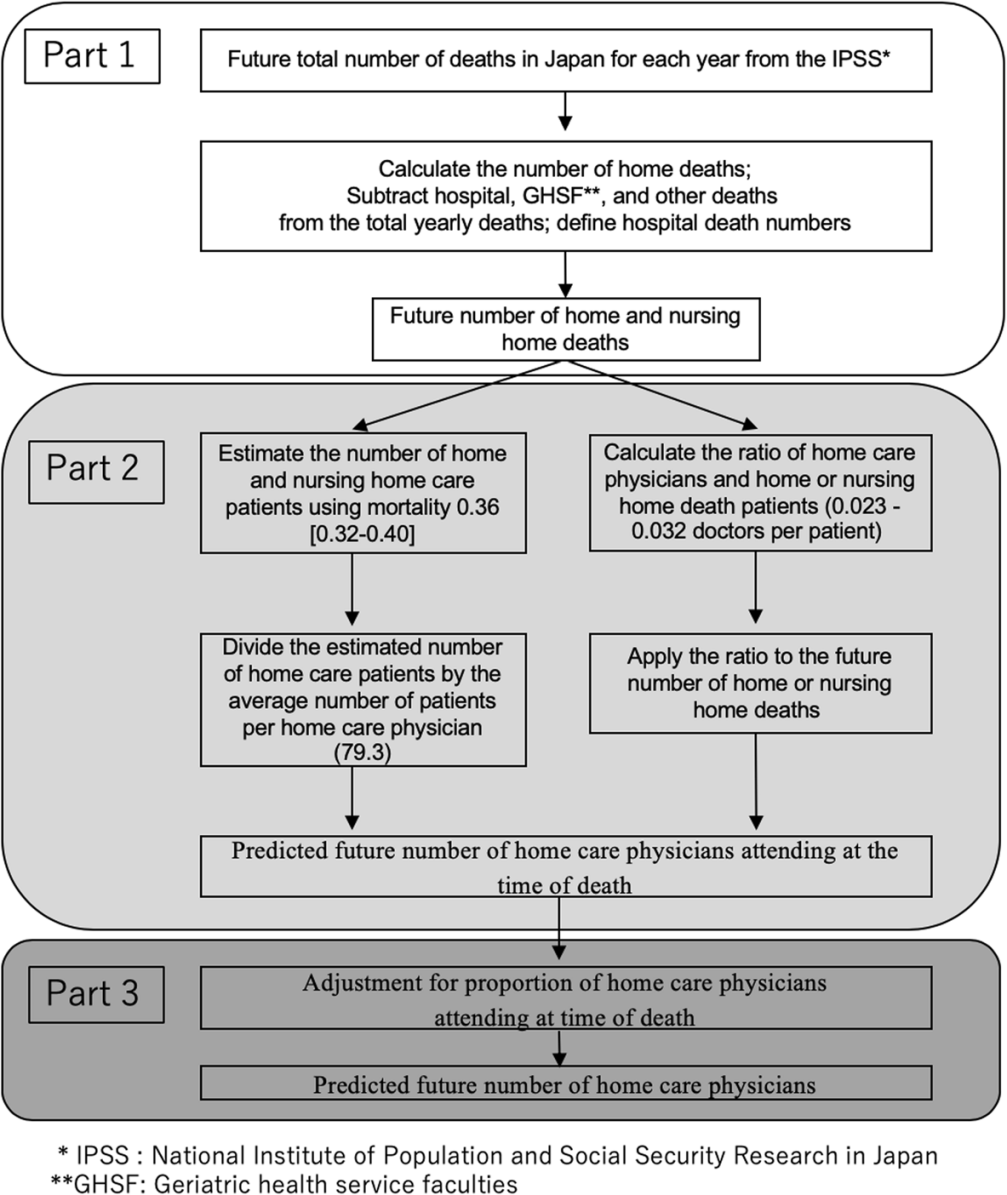
Study flow chart

The process for estimating future need for home care physicians is summarized in Fig. 1. Part 1 projects annual home and nursing home deaths based on estimated statistics from several official sources. Part 2 estimates the annual number of home care physicians who will provide end of life care in person using the figure derived in Part 1, by the following two methods:

- *Mortality method, Formula 1:* Dividing mortality into projected annual home and nursing home deaths gives the projected number of annual home or nursing home patients, which is then divided by the average number of patients per physician.
- *Physician-to-patient ratio method, Formula 2:* Using national survey data, the ratio of home care physicians to home or nursing home death patients is calculated and applied to projected annual home or nursing home deaths.

Finally, in Part 3, future home physicians divided by adjusted future proportion of home care physicians providing terminal care at home gives total future home care physicians.

### Part 1

We first calculated estimates of home and nursing home deaths. In 2017, the National Institute of Population and Social Security Research (IPSS) provided a prediction of future total deaths [13]. Annual IPSS reports consider current and past age, sex, birth, death, and immigration data, providing high, low, and mid-range projections of future deaths.

We calculated future home or nursing home deaths, subtracting deaths on hospitals beds including clinics and geriatric health service facilities (GHSFs), and others including accidental deaths. Importantly, the *Regional Healthcare Vision* aims to facilitate home medical care rather than increasing hospital beds, thus reducing hospital deaths. Hence, we applied three models in our projections. The first assumed total hospital deaths as constant after 2017, given that the Japanese government basically opposes additional hospital beds (pragmatic government model). The second assumed a constant proportion of deaths in hospitals, implying unchanged health policy with increases following current trends (non-intervention model). The third model assumed a 1.1% annual decrease in hospital deaths from 2018 to 2025, following the government’s reduction target from 1.31 million to 1.19 million beds by 2025 [14, 15]. This follows reports that available hospital beds correlate with hospital deaths [16]. However, hospital deaths are assumed to be constant after 2025 because of the growing elderly population (dynamic government model).

Government vital statistics in 2017 showed nursing home and GHSF deaths represent 7.5 and 2.5% of all deaths, respectively [17]. The Japan Cabinet study and Fukui et al. reported that at most 8.6 and 12% of elderly Japanese prefer nursing homes or GHSFs, respectively [7, 18]. To estimate the minimum number of home deaths, we set the combined proportion of nursing home and GHSF deaths at 12% of all future deaths, and other causes of death at a constant 2.05% using the 2017 vital statistics [17].

After projecting home and nursing home deaths, two methods were used. We must note that the MHLW counts only clinics and hospitals which can provide home care and the number of times it is provided, and not exact numbers of home care physicians and home or nursing home patients.

### Part 2

#### Mortality approach

The first method began by predicting future annual total home and nursing home patients by dividing deaths by mortality: 0.36 [0.32–0.40]/year. Mortality data were derived from a prospective Japanese multi-center home care cohort study [19]. Next, to predict the future number of FTE home care physicians, the estimated total number of patients receiving home and nursing home care was divided by the average yearly number of home care patients per physician (79.3) (Fig. 1, Formula 1). This was calculated as follows. The MHLW reported the average number of home care patients per HCSC (65.8) [20]. In addition, we calculated the FTE physician-HCSC institution ratio (0.83) from a MHLW survey of home health care [21]. Finally, home and nursing home care patient numbers were divided by average patients per home care physician. Nursing homes were included because many home care physicians also provide care in nursing homes. Thus, serving in a nursing home also qualifies as home care medicine in Japan. Moreover, most Japanese home care medicine is supported by clinics. Therefore, our study also included future home care physicians at clinics.

#### Physician-to-patient ratio approach

The second method uses the ratio of home deaths to FTE physicians shown in Fig. 1, Formula 2. The MHLW does not monitor exact annual numbers of home or nursing home care patients and physicians, but in 2014, it conducted its triennial survey of all medical institutions in Japan, gathering data from 1 month (September) [22]. We estimated both numbers based on the 2014 survey, which showed hospitals and clinics taking part in home care and numbers of patients who died under a physician’s care without incident (8996). We estimated the number of home care physicians using the home health care survey, which provided the average number of physicians for each type of clinic and hospital [21].

Thus, we assume the precise number of monthly home care physicians (3758) and consider it equal to the annual number of home care physicians. The number 3758 was calculated as follows. The MHLW institution survey showed that the numbers of clinics and hospitals whose physicians attended at patients’ deathbeds were 4312 and 476, respectively [22]. Although the deathbed numbers included both patients’ homes and nursing homes, the survey excluded nursing homes with adjacent institutions providing care. Further, nursing home death accounted for less than one-third (27.8%) of home and nursing home deaths in 2014 [17], and the numbers of physicians were surveyed for a 1-month period. Considering the relatively low ratio of deaths occurring at nursing homes, the short term of the study, and the fact that very few physicians from clinics or hospitals are likely to work exclusively at nursing homes, we regarded the numbers of physicians as those who attended patients’ deathbeds at home. Next, the ratios of FTE physicians to clinics and hospitals were calculated to be 0.75 and 1.1, respectively, from the MHLW home care medicine survey, although hospital data were from home care support hospitals (HCSH) only. This is because our literature search did not uncover any surveys or data for the other types of hospitals providing home care medicine, including the MHLW survey [21]. We regarded all hospitals providing home care medicine as HSCHs. Finally, these ratios were applied to the clinic and hospital numbers.

However, considering the ratio of home deaths to total deaths in September from the 2014 vital statistics, we estimate that 12,487 people died at home during this month [23]; thus, the medical institutions survey might underestimate the number of home deaths because the figures were not reported by medical staff [22]. Hence, we regard 5216 which is 3758 times 12,487/8996 as the highest number of home care physicians in 2014, with the annual number of home deaths being 162,599 [17]. Finally, our estimation uses a FTE physician-to-patient ratio between 0.023 and 0.032, which was applied to projected future home and nursing home deaths.

### Part 3

#### Adjustment for proportion of home death

After calculating the minimum future number of home care physicians, we considered the creation of training systems for home care physicians by adjusting for the proportion of patients whom physicians visited at the time of death at home, instead of in hospital.

Surprisingly, the state of Japanese home care medicine is such that physicians attended at their patients’ deathbeds (home death) at only 52.7% of institutions that provide home care medicine [11]. We calculated that the proportion of FTE physicians who attended at their patients’ deathbeds (home death proportion) was 55.0% in both Nomura et al. and the MHLW survey [11, 21]. Therefore, the minimum number of home care physicians should be about 1.82 times (1/0.55) the number who actually attend at deathbeds. This estimation can be confirmed in the national study [22], which reported that in 2014, 7998 home care institutions took care of patients until death, but also showed there were at least 20,597 clinics and physicians regularly providing home care during a one-month period. Furthermore, Nomura et al. implied that, with time, home care medicine will develop and improve, correlating with the increase in the proportion of home death patients whom physicians attend at their deathbeds [11]. Hence, we assumed that the home death proportion would gradually increase from 55.0 to 62.3% by 2040, a method which is unique to this study. This number (62.3%) is calculated as follows. There are four types of clinics providing home care in Japan: enhanced single center HCSCs, enhanced multi-center HCSCs, ordinary HCSCs, and other clinics [21]. Nomura et al. and the MHLW report the proportion of home death at the three types of HCSCs (77.8, 88.3, and 59.3%; 68.9, 57.5, and 48.3%) and the number of each type of institution that participated in the studies (54, 240, and 563; and 10, 61, and 309), and the MHLW study reported the FTE physician-to-clinic ratio for home care medicine for each of the three types of HCSCs (2.4, 1.2, and 0.7) [11, 21]. We multiplied the number of each of the three types of HCSC physicians and each study’s home death proportion and averaged the results. Next, the averaged result was divided by the total number of HCSC physicians. Finally, we averaged the two proportions.

The formula is as follows:

$$ HCSC\ physicians\ home\ death\ proportion=\left(\ \frac{\Sigma\ \mathrm{number}\ \mathrm{of}\  HCSC\ physicians\times proportions\ of\ home\ death s\ }{Total\ number\ of\ HCSC\ physicians}\right) $$

$$ Average\ HCSC\ home\ death\ proprtion=\left( Average\ HCSC\ physicians\ home\ death\ proprtions\ from\ both\ studies\right)/2 $$

## Results

**Fig. 2:**
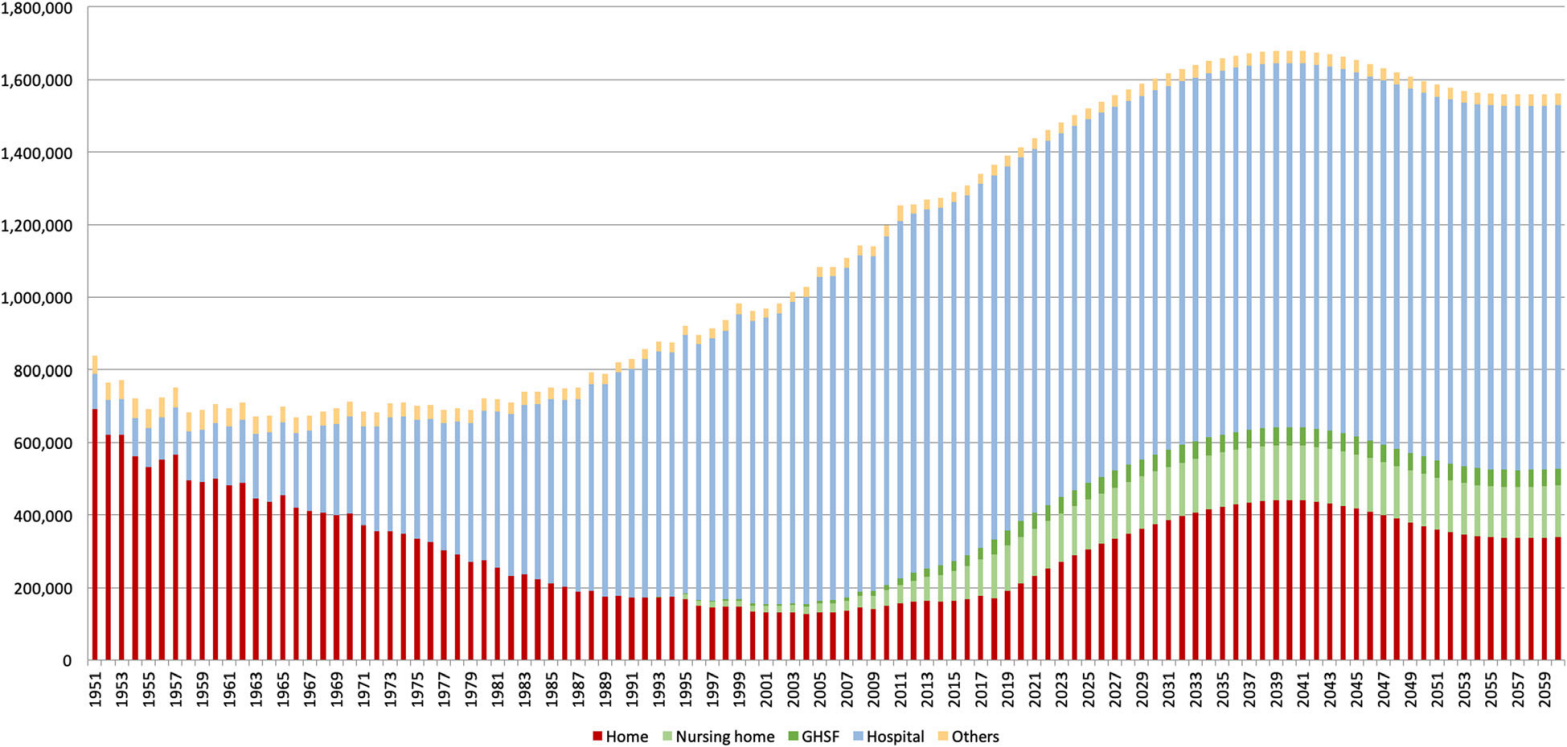
Deaths by location past records (1951–2017) and future predictions (2018–2060)

Past death numbers and locations, which are collected as a national vital statistic with 99.9% of death records, can be seen in Fig. 2 (1951–2017) [17]. In the 1950s, most people in Japan died at home, though the proportion decreased until the early twenty-first century, while hospital deaths increased rapidly. Previously, Japan had nursing homes and GHSFs, which are essentially short-term nursing homes. After 1994, the government began counting nursing homes and GHSFs, and both have increased gradually.The future projection shows the pragmatic government model (constant hospital deaths), suggesting that home and nursing home deaths will rise from 340,000 in 2020 to just below 600,000 until 2040 and then begin trending downwards to 480,000 in line with total deaths.

**Fig. 3:**
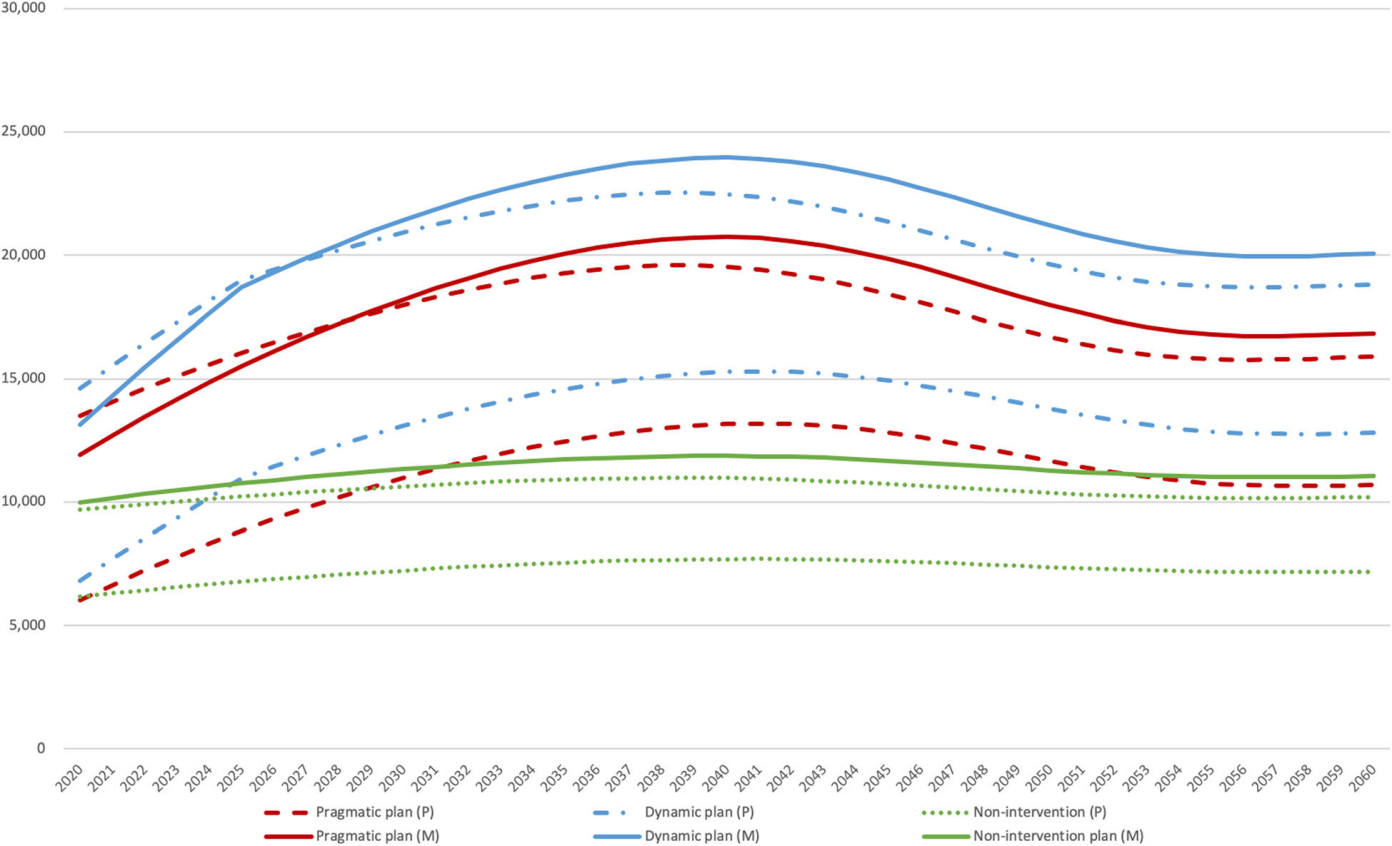
Home care physicians 2020 to 2060, mortality and population method

In our projections, a similar trend is seen in the dynamic government model. The number of deaths will rise steeply from 370,000 in 2020 to 530,000, and keep rising to 680,000 until 2040, when it will begin gradually decreasing to around 570,000. On the other hand, if the proportion of hospital deaths is steady until 2060, indicating no change in government health policy (non-intervention plan), the number of home care or nursing home care patients will stay constant at around 300,000. The trends found in the 3 models parallel each other quite closely, as shown by the bold lines in Fig. 3. (Additional file 3).

### Projected need for home care physicians

#### Mortality method (health needs approach)

Based on projected numbers of at home and nursing home deaths, the number of home care physicians attending at the time of death calculated using mortality (mid-range) in Fig. 3 (bold lines) shows that demand will decrease gradually after 2040, before stabilizing. The pragmatic and dynamic government models both show over 20,000 home care physicians attending at the time of death by 2040, while the non-intervention model projects a steady trend hovering around 11,000. Additionally, among the mortality method estimates (high, mid, and low range), the differences between the high-mid and mid-low estimates in 2020 are about 4500 and 3500, respectively. Thereafter, the differences will gradually decrease to 2500 and 2000, respectively, by 2060. (Additional file 4).

Figure 3 (dotted lines) also reveals a similar up-and-down trend projected using the physician-to-patient method, suggesting that in 2040, the pragmatic and dynamic government models imply a need for 2–2.5 times more home care physicians than the non-intervention model.

### Projection of total number of home care physicians after final adjustment

**Fig. 4:**
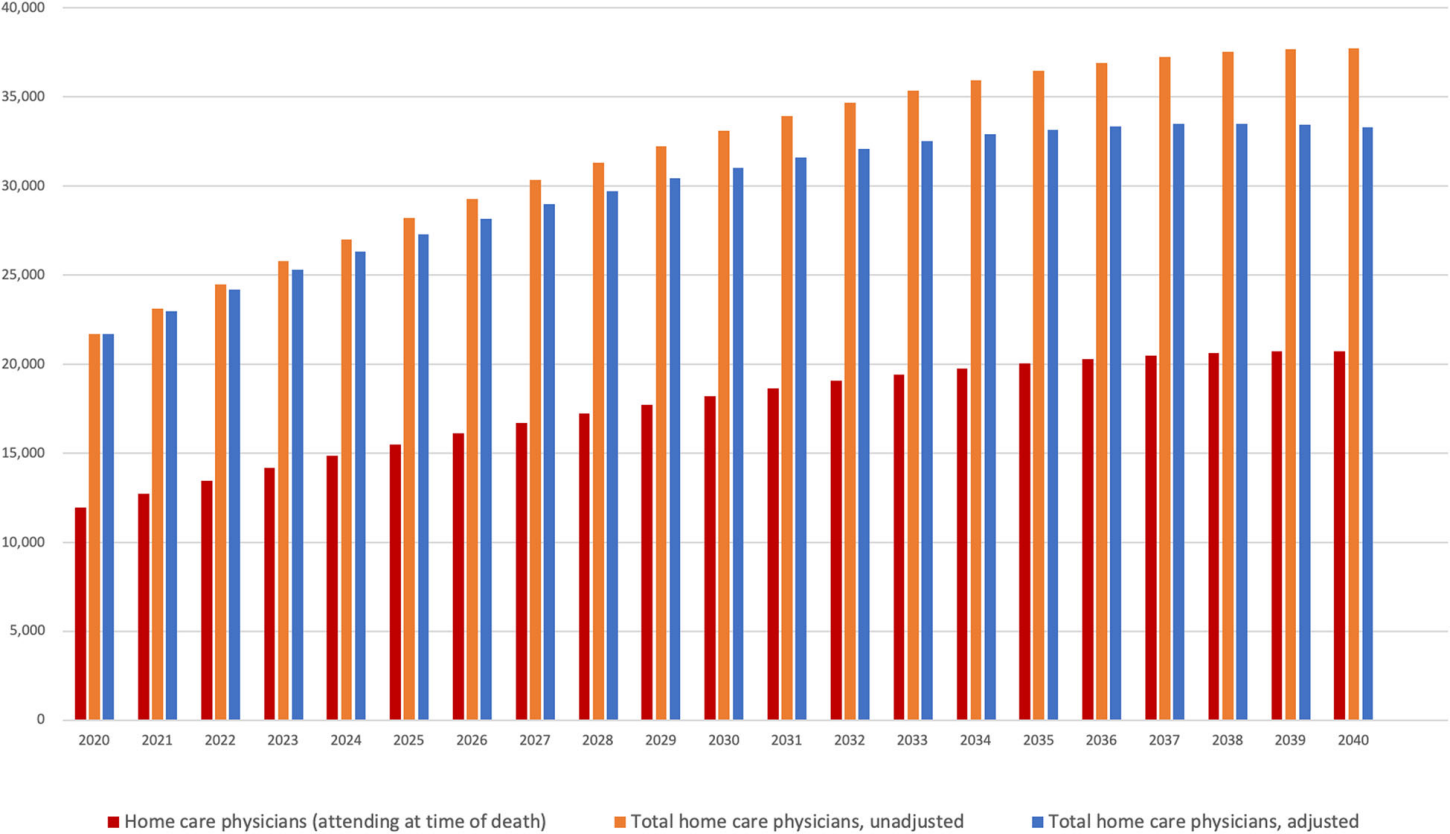
Future need of Home Care Physicians mortality method with and without adjustment for proportion to home deaths

Our final results are shown in Fig. 4, which illustrates minimum and total home care physicians using the mid-range death estimate and the mortality method with and without adjustment for the proportion of home deaths (55.0% at present, 62.3% in 2040). Demand for physicians will rise year by year without considerable development of home care medicine, but with improvement, the need for physicians will stabilize after the late 2030’s. The current number of home care physicians is not precisely known because rather than directly counting home care physicians and patients, the Japanese government records numbers of clinics or visits to patients’ homes. However, it is estimated to be around 19,500 FTE physicians (16,500 at clinics and 3000 at hospitals) and 43,500 physicians by head count (35,000 at clinics and 8500 at hospitals). Nomura et al. reported that in 2017, 4386 clinics represented around 20% of all Japanese clinics providing home care medicine, implying that there were 21,930 clinics providing home care medicine [11]. The FTE physician-to-clinic ratio was 0.75 from the MHLW survey [21], suggesting that there are 16,448 FTE home care physicians at all clinics. Moreover, the MHLW survey shows 2702 hospitals gave regular home care service in September of 2017 [24]. Because regular home care visits by physicians happen twice a month in Japan, we regard 2702 as the annual number of hospitals providing home care medicine. After calculating that the total number of FTE home care physicians per-hospital is 1.1 based on the MHLW survey [21], there appear to be about 3000 (2972) home care physicians at hospitals.

The physician head count was estimated similarly. The Nomura et al. survey showed the number of physicians (head count) per-clinic was 1.6, suggesting that there are 35,088 home care physicians (head count) at all clinics [11]. The number of home care physicians (head count) per-hospital was estimated to be 3.1 based on MHLW survey [21]. Although this 3.1 was calculated only from the number of full-time physicians (head count) providing home care medicine in HCSHs, we regard it as a head count of both full- and part-time physicians because the number of part-time physicians providing home care medicine was likely very low. Hence, there appear to be about 8500 (8376) home care physicians at hospitals. Furthermore, the physician (head count) to clinic ratio of 1.6 in the Nomura et al. survey and the FTE physician to clinic ratio of 0.75 in the MHLW survey enable us to convert FTE physicians to head count, using a multiplication factor of roughly 2.13 (1.6/0.75) [11, 21].

### Sensitivity analysis

**Fig. 5:**
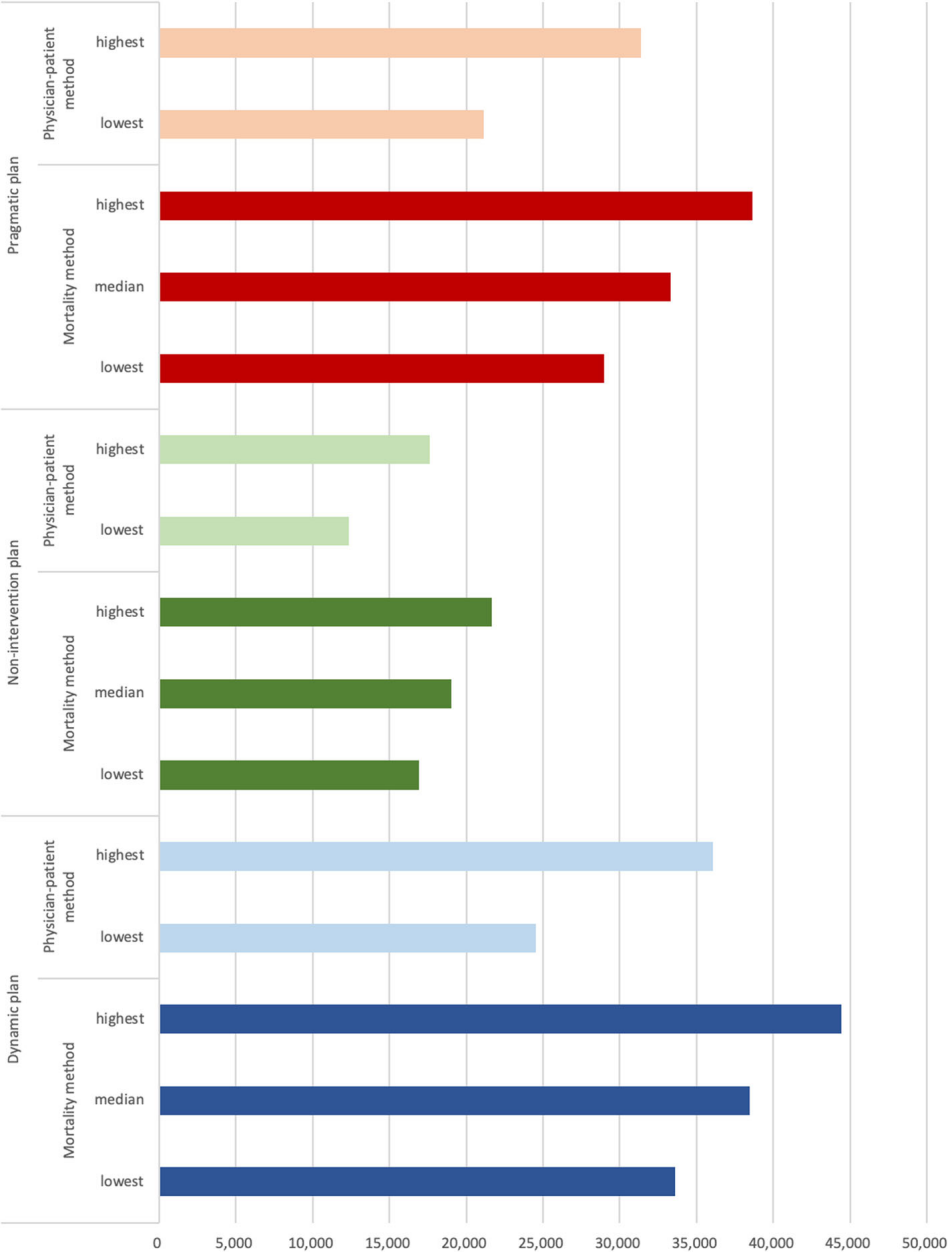
Home Care Physicians in 2040

For sensitivity analysis, we compare projected numbers of home care physicians in 2040 in Fig. 5. There was overlap between the mortality method and physician-to-patient method projections. In addition, the present (2017) number of FTE home care physicians is estimated to be around 19,500 (including 16,500 at clinics). This is slightly above but very near our mortality method’s (mid) estimation of 17,666 in 2017. We regard this difference as within an acceptable range because our estimations were calculated based on physicians providing home care at HCSCs, who are expected to have specialized training and education, as well as support staff, enabling them to take care of more patients compared with ordinary physicians. In contrast, the present number of total home care physicians includes those who provide home care as a minor part of their jobs at ordinary clinics or hospitals.

## Discussion

We found that by the late 2030’s, Japan will need approximately 14,000 additional FTE home care physicians, or 28,000 by head count, which are 1.72 and 1.64 times the current numbers, respectively. However, our study also shows that developing home care may help suppress the growing overall need for physicians. Further, demand for home care physicians will diminish after 2040.

The estimation of future hospital deaths was based on three assumptions: the pragmatic and dynamic government models, and the non-intervention model. Among these, the non-intervention model is unrealistic considering dropping hospital admission statistics including total hospitals, total beds, and average stay length. The dynamic government model might underestimate the number of hospital deaths because of the steadily increasing aged population despite the whole population shrinking and the present and future longest life expectancy in the world until the year 2100, which implies a constant need for hospital beds [1]. Considering this, we believe that the pragmatic model is the most realistic projection. Hence, in order to supply sufficient home health care smoothly while preparing for the growing aged population, our results suggest that the Japanese government must increase the number of skilled home care physicians. However, the results also suggest that demand for home care physicians will decrease long-term. Therefore, reduction in home care physician training should be considered in the 2030’s.

There are four conventional methods to estimate future health workforce requirements: the health workforce-to-population ratio method, the health needs method, the service demands method, and the service targets method [12, 25, 26]. Furthermore, other more sophisticated approaches have been advanced recently. As an example, the cohort-component model considers physicians’ ages as well as geographical and other factors, and has been implemented in physician workforce forecasting [27]. System dynamics models and geographic information technology have been implemented in other analyses [28].

Our analysis employed the first two methods. Our physician-to-patient ratio method is a variant of the health workforce-to-population ratio method. This method is simple, straightforward, and widely used [29]. However, errors or miscalculations of data for the baseline year influence outcomes for the target year. Second, our mortality method, using epidemiological data, is a modification of the health needs method. The method is logical, straightforward, and consistent with professional ethics [26]. However, it has some disadvantages, one of which is that the data needs to be updated to reflect changes in technology and health care services. Therefore, we adjusted the home death proportion considering that future home care physicians will probably concentrate more on home care and its training, suggesting a greater proportion of physicians will work at HCSCs instead of ordinary clinics as we approach 2040, in accordance with the MHLW *Regional Healthcare Vision* reinforcing home care medicine and its future development in Japan [16]. While we believe that our adjustment of the home death proportion will reflect various factors such as improvement in home care medicine, it is based on assumptions and limited to only one aspect of home care.

Home care medicine is a primitive but developing field of heath care. The concept of home care medicine may appear superficially inefficient and regressive. While there is variation among the definitions and systems of home care medicine, as well as the roles and contributions of physicians, home health care is gaining attention in many countries. Physician’s home visits are core tasks of the family physician in Germany and Belgium [30]. Furthermore, the problems of an ageing society are not peculiar to Japan and industrialized countries; the elderly population in developing regions is increasing more rapidly than in developed regions [2]. While many medical workforce analyses have covered physicians, primary care physicians, surgeons, specialists, and nurses, we are aware of no published reports projecting home care physicians at the time of this writing. Therefore, applicable workforce analysis of home care physicians is also warranted in other countries. Our study employed relatively simple methods with potential for broad application. While our mortality method requires mortality data for a country, our study shows that the physician-to-patient method, which requires only a count of home deaths and home care physicians, is simple and produces valid estimation compared with the mortality method. If more attention is paid to home care, the potential benefits could extend beyond Japan.

Although our methods depend on some assumptions, our estimations are highly reliable. This is evidenced by the small difference between our mortality method mid-range estimation and the present number of home care physicians in 2017, which are reached through wholly different methods. Where the mortality method is based on the number of future home deaths and mortality, the present number of home care physicians is based on counts of clinics and hospitals and physicians per institution. In addition, our mortality method used credible home care patient data from a previous multi-center study [19] with a sufficiently narrow 95% confidence interval. Furthermore, our results agree with past studies forecasting numbers of physicians, which anticipated increases into the 2030’s [27, 31]. We are therefore convinced that our projections are not unreasonable.

There are some limitations to our study. First, our estimations were limited by the accuracy of available data, including the fact that the future nursing home death number was based on patient preferences [18]. Also, our mortality data comes from an interim analysis presented at an annual conference, although we believe the figures are precise [19]. Second, while our two methods produced similar projections, which suggests valid estimations, it is difficult to completely account for present and future variability, such as drastic changes in health policy and natural calamities. Our results depend on the average yearly number of home care patients per physician, which can vary over time and according to public policy. Geographical disparities and other variables such as future development of nurse practitioners affect the number of physicians, with wide gaps between physician-rich and physician-poor areas [27]. Although we estimated the future number of physicians based on FTE and converted to head count, the precise proportion of full-time and part-time physicians and variations in individual contribution to home care medicine should be analyzed separately. Finally, practical constraints required us to make several assumptions in applying the available data. For example, we applied the physician-to-patient ratio from home deaths in 2014 to future home and nursing home deaths. With accurate nursing home physician data, we could estimate more precisely. Further, while the mortality approach mainly focuses on home care support clinics, the physician-to-patient ratio approach includes home care support and ordinary clinics. All of these limitations should factor into further studies projecting numbers of home care physicians.

## Conclusion

Our findings indicate an increasing need for home care physicians through 2040, anticipated to rise to at least 1.6–1.7 times the current number. However, demand for these physicians will decrease, then stabilize by 2060. The improvement of home care medicine could positively influence changes in the physician workforce.

## Supplementary information

**Additional file 1.** Visual study flow chart. Graphic representation of the methods used in this study.

**Additional file 2.** Baseline data summary table. Summary of baseline data sources.

**Additional file 3.** Projection of home and nursing home deaths (mid-range) from 2020 to 2060 for three plan types. Line graph depicting mid-range projections based on three plan types.

**Additional file 4.** High, mid, and low estimates of future home care physicians (full-time-equivalent) attending at patients’ deathbeds in the pragmatic plan. Line graph depicting high, mid, and low estimates of future FTE home care physicians attending at patients’ deathbeds based on the pragmatic plan.

## Data Availability

The datasets complied, generated, and analyzed in the present study are available from the corresponding author upon reasonable request.

## Abbreviations

MHLW: The Ministry of Health, Labour and Welfare in Japan
FTE: Full-time equivalent
IPSS: National Institute of Population and Social Security Research in Japan
GHSF: Geriatric health service faculties
HCSC: Home care support clinic
HCSH: Home care support hospital
