# Supplementary figures and images for: The need for home care physicians in Japan – 2020 to 2060
Source: BMC Health Serv Res. 2020 Aug 15;20:752. doi: 10.1186/s12913-020-05635-2 (PMC7429680; doi:10.1186/s12913-020-05635-2)

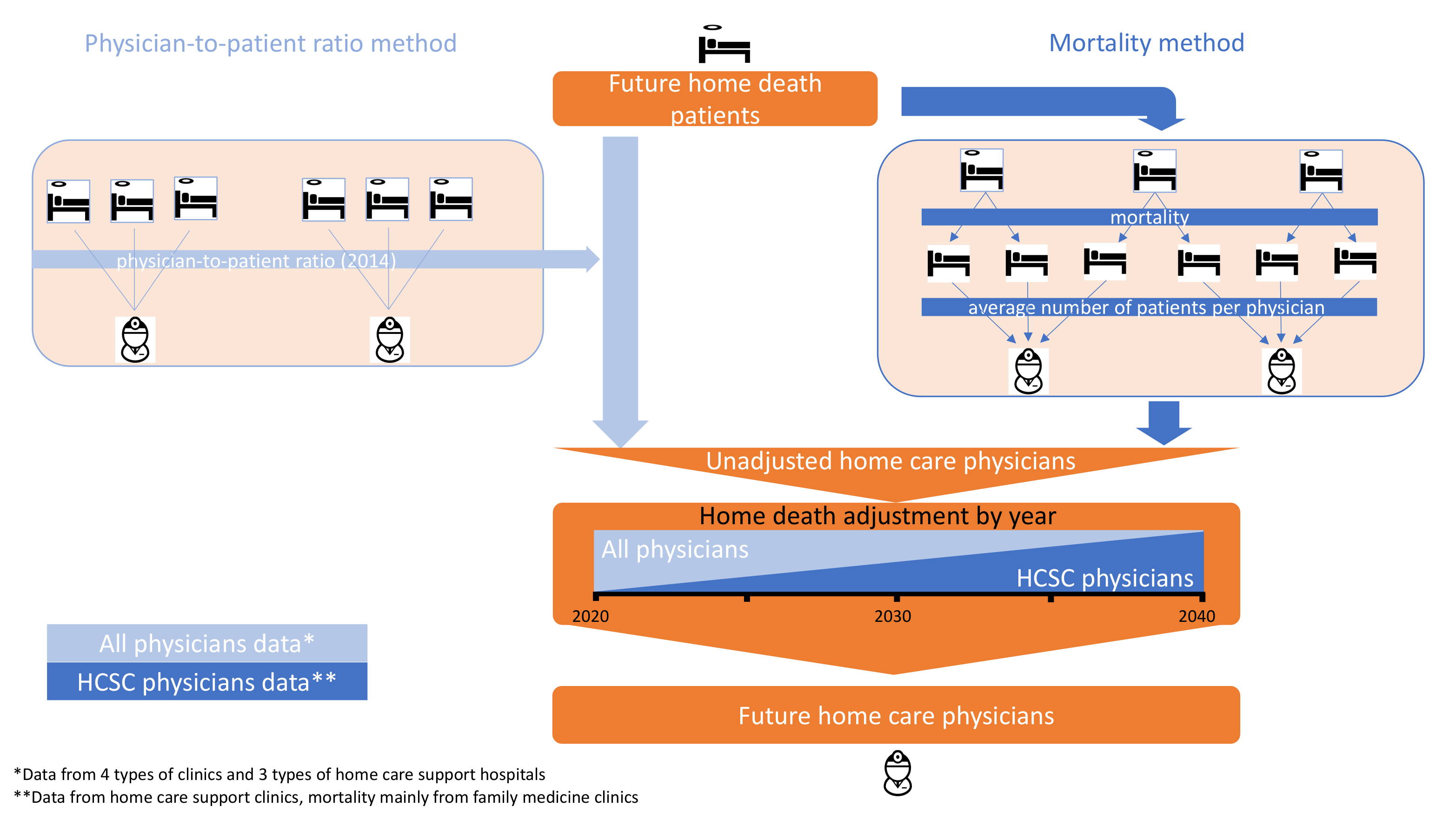

Supplement: Supplementary file 1 — Additional file 1. Visual study flow chart. Graphic representation of the methods used in this study. [file 12913_2020_5635_MOESM1_ESM.png]

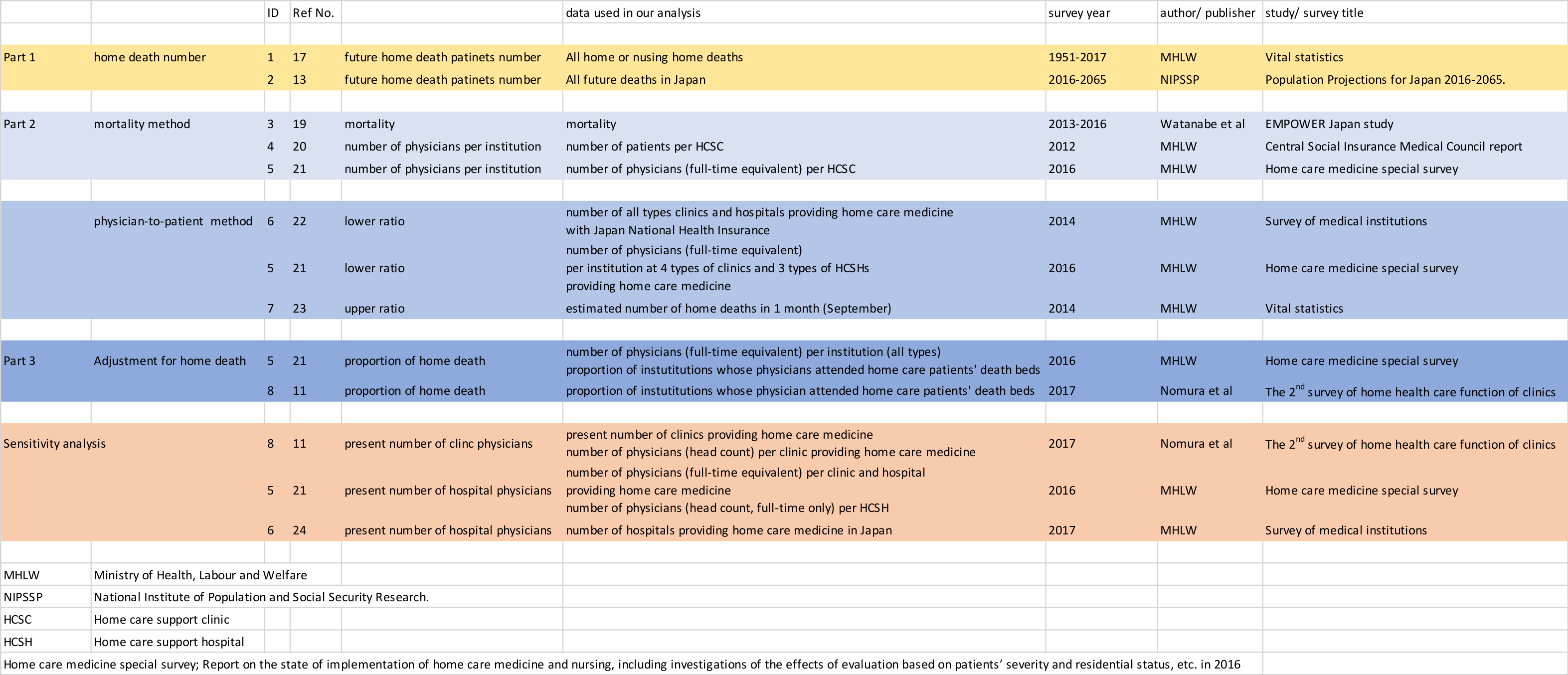

Supplement: Supplementary file 2 — Additional file 2. Baseline data summary table. Summary of baseline data sources. [file 12913_2020_5635_MOESM2_ESM.png]

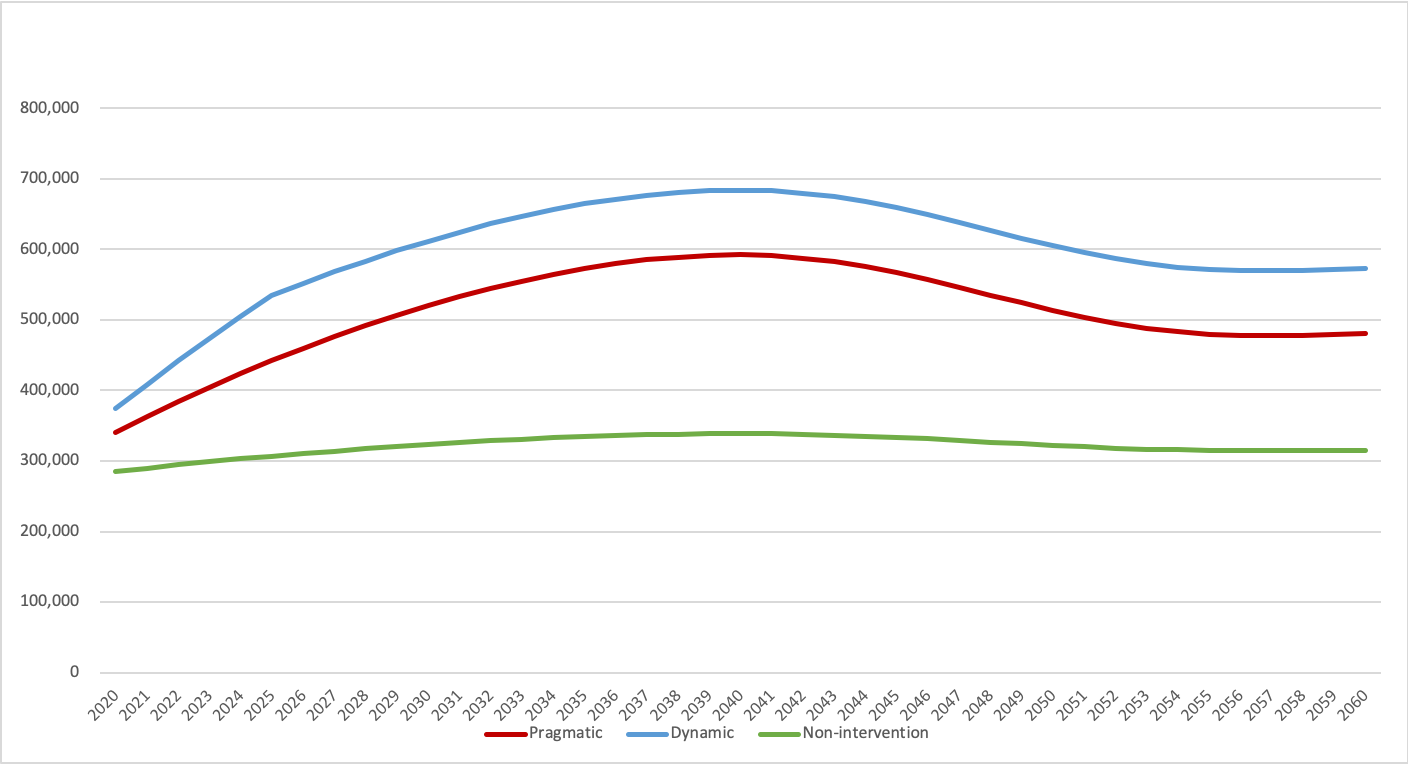

Supplement: Supplementary file 3 — Additional file 3. Projection of home and nursing home deaths (mid-range) from 2020 to 2060 for three plan types. Line graph depicting mid-range projections based on three plan types. [file 12913_2020_5635_MOESM3_ESM.png]

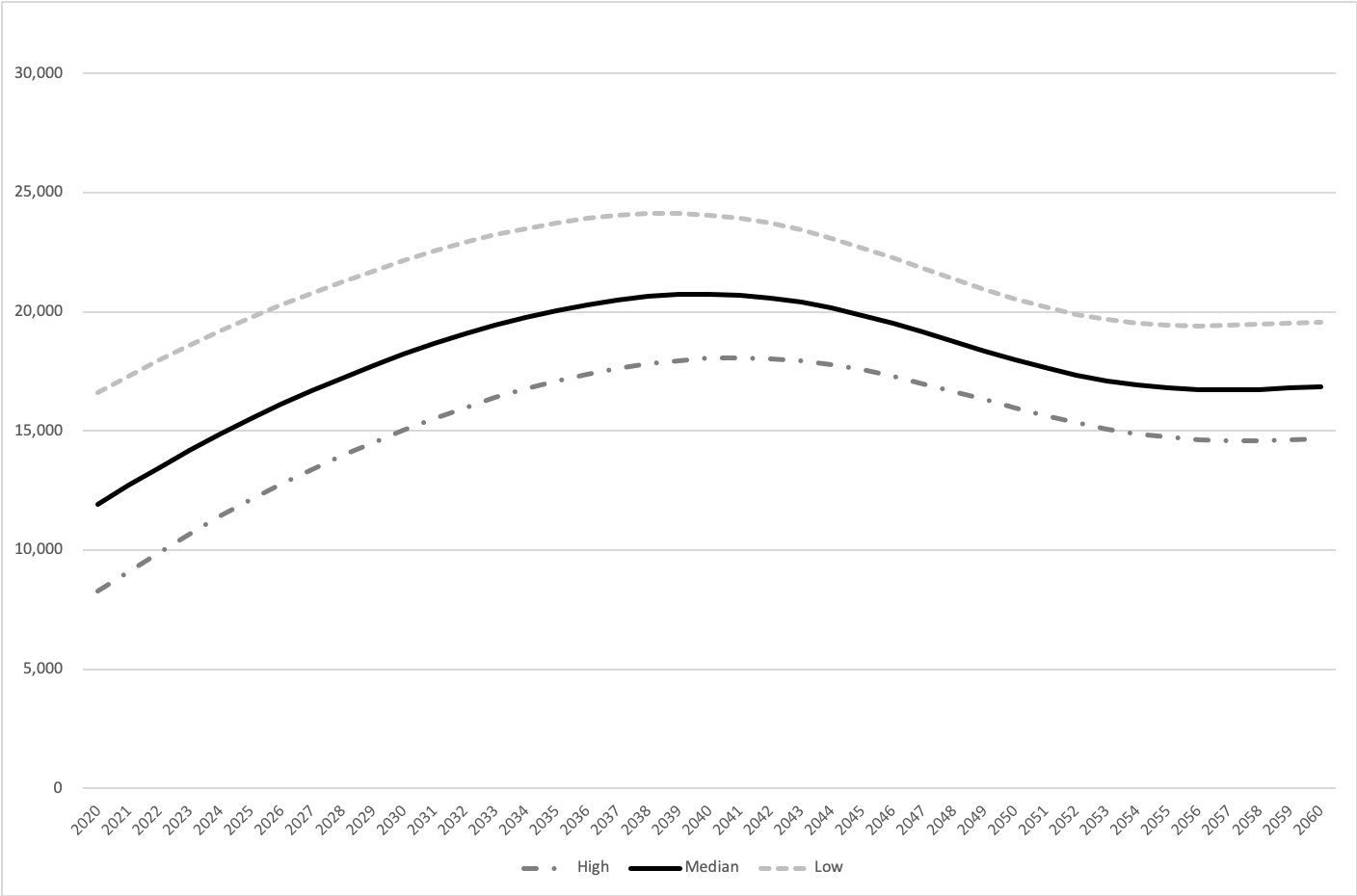

Supplement: Supplementary file 4 — Additional file 4. High, mid, and low estimates of future home care physicians (full-time-equivalent) attending at patients’ deathbeds in the pragmatic plan. Line graph depicting high, mid, and low estimates of future FTE home care physicians attending at patients’ deathbeds based on the pragmatic plan. [file 12913_2020_5635_MOESM4_ESM.png]
